# Development of a brain MRI-based hidden Markov model for dementia recognition

**DOI:** 10.1186/1475-925X-12-S1-S2

**Published:** 2013-12-09

**Authors:** Ying Chen, Tuan D Pham

**Affiliations:** 1 Aizu Research Cluster for Medical Engineering and Informatics Research Center for Advanced Information Science and Technology. The University of Aizu Aizuwakamatsu, Fukushima 965-8580, Japan

**Keywords:** Dementia, Hidden Markov Model (HMM), Classification, Magnetic Resonance Imaging (MRI), Alzheimer's Disease (AD), Regularity Dimension, Semi-variogram, Vector Quantization (VQ)

## Abstract

**Background:**

Dementia is an age-related cognitive decline which is indicated by an early degeneration of cortical and sub-cortical structures. Characterizing those morphological changes can help to understand the disease development and contribute to disease early prediction and prevention. But modeling that can best capture brain structural variability and can be valid in both disease classification and interpretation is extremely challenging. The current study aimed to establish a computational approach for modeling the magnetic resonance imaging (MRI)-based structural complexity of the brain using the framework of hidden Markov models (HMMs) for dementia recognition.

**Methods:**

Regularity dimension and semi-variogram were used to extract structural features of the brains, and vector quantization method was applied to convert extracted feature vectors to prototype vectors. The output VQ indices were then utilized to estimate parameters for HMMs. To validate its accuracy and robustness, experiments were carried out on individuals who were characterized as non-demented and mild Alzheimer's diseased. Four HMMs were constructed based on the cohort of non-demented young, middle-aged, elder and demented elder subjects separately. Classification was carried out using a data set including both non-demented and demented individuals with a wide age range.

**Results:**

The proposed HMMs have succeeded in recognition of individual who has mild Alzheimer's disease and achieved a better classification accuracy compared to other related works using different classifiers. Results have shown the ability of the proposed modeling for recognition of early dementia.

**Conclusion:**

The findings from this research will allow individual classification to support the early diagnosis and prediction of dementia. By using the brain MRI-based HMMs developed in our proposed research, it will be more efficient, robust and can be easily used by clinicians as a computer-aid tool for validating imaging bio-markers for early prediction of dementia.

## Introduction

Dementia is an age-related neurodegenerative disorder but the cause is still essentially unknown. Alzheimer's disease (AD), the most common form of dementia, is characterized by loss of neurons and synapses in the cerebral cortex and certain subcortical regions. The current clinical diagnosis of AD is still based on clinical observation, neurological and neuropsychological testing. Advanced medical imaging techniques such as magnetic resonance imaging (MRI), positron emission tomography (PET), and single photon emission computed tomography (SPECT) have shown promise as non-invasive diagnostic indicators for AD that may lead to proposal of new diagnostic criteria [1,2]. Specially, volumetric MRI proves less expensive than other imaging methods and related studies have documented reductions in the size of specific brain regions in people with dementia as they progressed from mild cognitive impairment to severe AD [3,4]. However, it is still challenging to apply fully automated MRI analytic methods to identify potential AD neuroimaging bio-markers.

Studies involving brain MRI scans have tried to extract the most informative features for diagnosing dementia. Regions-of-interest (ROIs) analysis mainly focuses on brain structural changes in specific anatomical regions such as hippocampal [5,6], entorhinal [7,8], frontal [9-11], temporal [6,12], and parietal cortex [6,13] during disease progression. They provide valuable information of histopathological changes but somehow suffer from limitations like expert-dependency, region-limited, and time consumption. Multiple regions or the whole brain give more accuracy for dementia prediction since they extract typical features from the whole brain and are able to capture early signs of cognitive impairment before the onset of dementia. These studies include the measure of voxel-based differences in cortical volume [10,14], density [12], and thickness [13,15], etc. Cortex architecture such as sulcal folds and irregularity can be another important aspects worth studying since sulcal folds are the principal surface landmarks of the human cerebral cortex, and exhibit structurally complex patterns [16] that are postulated to reflect underlying connectivity [17]. However, very few studies have reported the changes of cortical architecture in dementia. In our previous study, we have quantified the cortex structure complexity using entropy method [18], and found a significant higher global cortical structure complexity in AD subjects compared to cognitive normal. This increase was also found to be accompanied with aging. These features have the potential to serve as sensitive surrogate markers and are capable of quantifying the extent of brain degeneration in dementia. However, modeling that can best capture brain structural variability and can be valid in both disease classification and interpretation is extremely challenging.

For dementia diagnosis, multivariate classification techniques have been proposed in literatures such as support vector machines (SVM) [19,20], artificial neural network (ANN) [21], and decision trees (Trees) [22]. Hidden Markov Models (HMMs) is a popular double stochastic model which is able to extract the underlying statistics using a compact set of features [23]. HMM is especially suitable for sequence data modeling compared to other conventional classifiers. It has been applied in white matter hyperintensities quantification [24], spatio-temporal analysis of brain MR images [25] and age prediction [26]. In the current study, we aimed to establish a computational approach for modeling the MRI-based structural complexity of the brain using the framework of HMMs for dementia recognition. Regularity dimension and semi-variogram was used to extract structural features of the brains. Regularity dimension is based on sample entropy which quantifies the system complexity. Semi-variogram can estimate the spatial distribution of the system. Vector quantization (VQ) method was applied to convert extracted large feature data to small prototype data. The output VQ indices were then utilized to estimate parameters for HMMs. Classification was carried out using a data set including both non-demented and demented individuals with a wide age range.

## Materials and Methods

### System architecture

**Figure 1:**
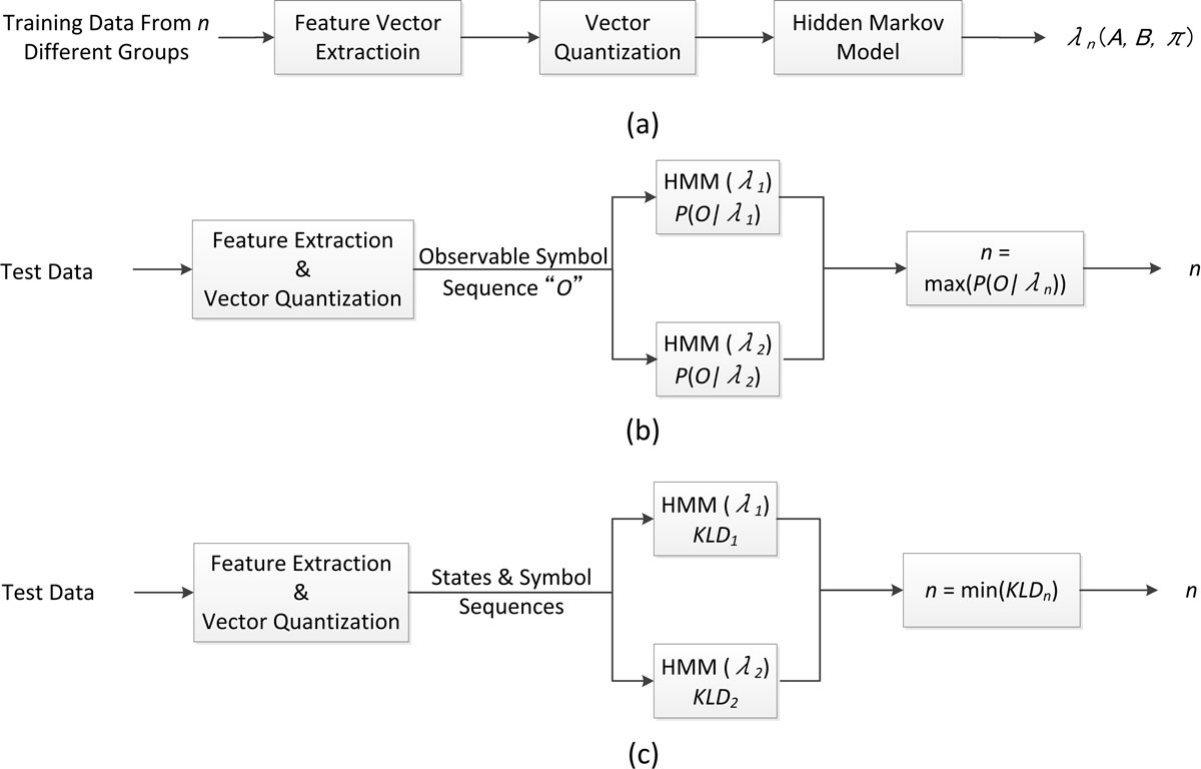
**System architecture**. Hidden Markov Models (a) training, and (b)(c) testing.

Two fundamental steps are required to design the classifier. The first one is to extract features that can best characterize and discriminate between different group of subjects, and the second is to select an appropriate classifier paradigm. Figure 1 shows the architecture of the proposed classification system. In the current study, two features were extracted using regularity dimension and semi-variogram separately from time series generated from each MRI slice. Two feature sequences were then obtained from time series of subsequent MRI slices over the whole brain. VQ allows each feature sequence to be represented as index sequence which can be defined as state or observable symbol in HMM construction. Evaluation of the classifier includes training and testing stages. We used n-fold and leave-one-out cross-validation in model testing.

During the training stage, feature vectors were obtained from all training data from two different groups. The model parameters were estimated and optimized by using state and observation sequences generated from VQ. Therefore, each group will have one representative HMM (Figure 1(a)).

Two approaches were applied in HMMs testing. The first is to match the observed symbol sequence of test data against two different group HMMs previously obtained in the training stage and compute the probability of the sequence generated from the model separately. The test data which indicates a subject will be classified to the group which attains higher probability (Figure 1(b)). The second approach is to construct a HMM for each test data and compare it with group HMMs separately using Kullback-Leibler divergence (KLD). The test data will be classified to the group which has smaller KLD indicating greater similarity between the two models (Figure 1(c)).

### Subjects

All subjects were drawn from the Open Access Series of Imaging Studies (OASIS) database (http://www.oasis-brains.org/) [27]. From the database, we selected a data set consisted of a cross-sectional collection of young, middle-aged, non-demented and AD elder adults (Table 1). The subjects were all right-handed and included both men and women. Among the subjects, 75 of them had been clinically diagnosed with very mild to mild AD (with Clinical Dementia Rating (CDR) score of 0.5 or 1); and others with CDR score of 0. All studies were approved by the Institutional Review Board (IRB) of Washington University. Informed consents were obtained from all subjects at the time of study participation.

**Table 1:** Subjects Demographics and Dementia Status

	Young	Middle-aged	Elder (non-demented)	Elder (AD)
Number	75	75	75	75
Sex (female/male)	43/32	43/32	49/26	43/32
Age (years) CDR	20.2 *± *1.2 (18*∼*22) 0	46.0 *± *8.1 (30*∼*59) 0	74.5 *± *8.4 (61*∼*91) 0	77.3 *± *7.6 (62*∼*92) 0.5, 1 or 2

Values given are mean *± *SD.

Values in parentheses represent the range.

### MRI acquisition and data preprocessing

All images were corrected for inhomogeneity prior to further segmentation. For each subject, a grey/white/Cerebrospinal Fluid (CSF) segmented image [28] in which each voxel has been labeled as GM, white matter (WM), or CSF is provided. All images are in 16-bit Analyze 7.5 format, and are normalized into 176x208x176 voxel-wise images. Additional details of the image characteristics can be found at (http://www.oasis-brains.org/) [27].

### Time series extraction

In order to generate 1-D signals that allow the application of regularity dimension, the surface structure of the GM should be represented by time-series. For each MRI slice, the time series represent the distances measured from subsequent outer boundary points to the GM center of mass. In semi-variogram analysis, the spacial locations of each point on the outer boundary were also included.

For each MRI slice, the center of the GM was detected by using the Matlab function called "regionprops" which calculates the centroids of the image regions labeled in the matrix of a two-dimensional array of nonnegative integers that represent contiguous regions. Each centroid is 1-by-n dims vector that specifies the center of mass of the region. The locating of the boundaries of GM in each 2-D scan was carried out by using the Matlab function called "bwtraceboundary" which traces the outline of an object in a binary image. All of these functions are available in the Matlab Image Processing Toolbox. The distances from consecutive points on the outer boundary to the center of the GM are then calculated. The distances are measured within each single MR slice (in a 2-D plane); one MR slice yields one time series. The whole cortical surface structure is then represented by 130-140 time series extracting from their corresponding MR image slices. For each individual, a sequence consist of 130-140 time series generated from the whole brain MRI slices can be obtained. The orders of the sequences are the same, from top to bottom.

**Figure 2:**
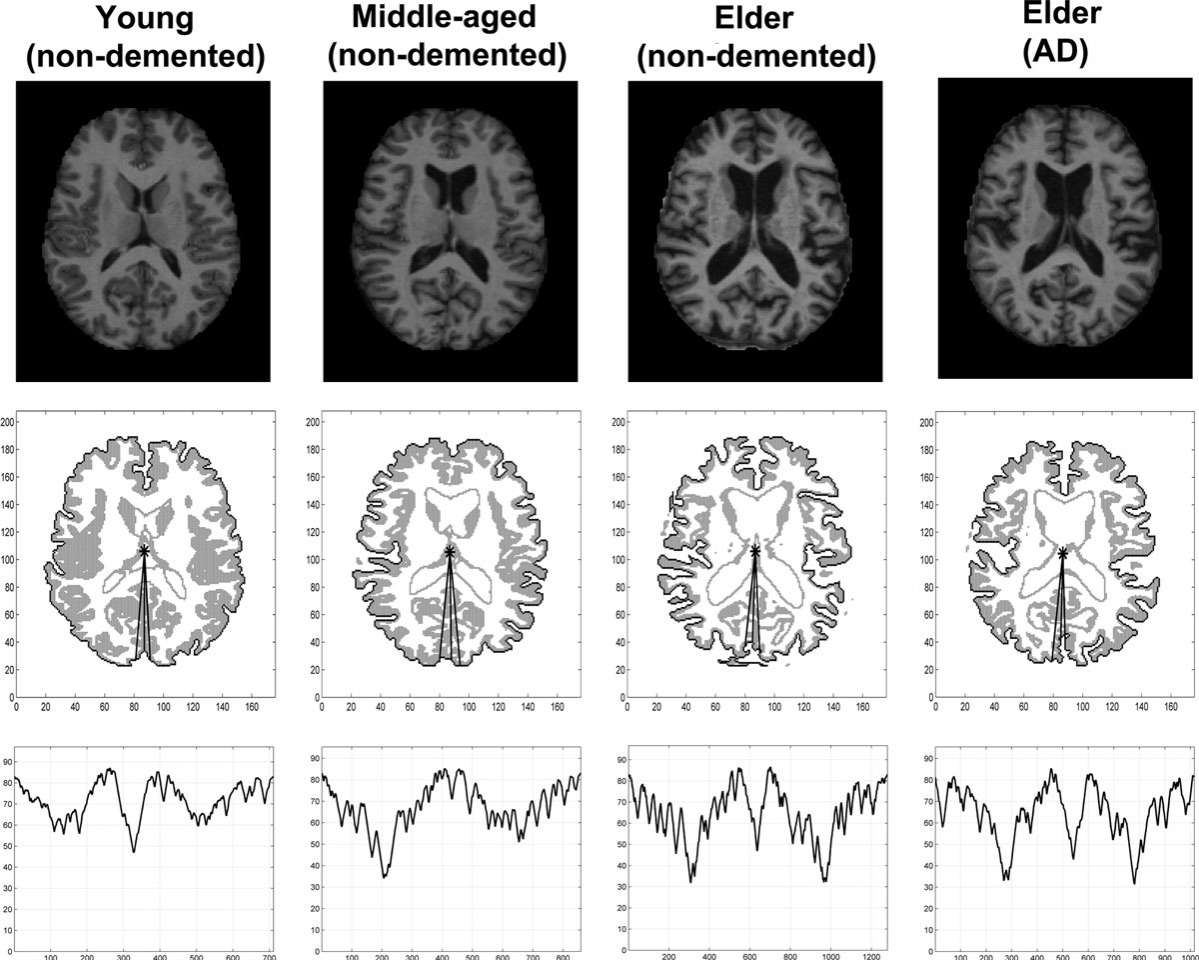
**Typical example of brain MR image for each group**. Typical examples of brain MR image for each group. Original image after removing the skull (the first row), detected boundary contours (black) of the grey matter (grey) for the same image (the second row), and the corresponding generated time series (the third row). In the third row of the figure, the time series indicates the distances measured from the grey matter center to subsequent outer boundary points.

By depicting the distances between the boundary points and the GM center of each cortical sheet, we were able to build up time series that can best reflect the micro-structures of the cortical surface architecture including its folding patterns and any shape changes, and as thus enable complexity analysis. Figure 2 presents typical examples of brain MRI, detected boundary contour of the GM and its corresponding generated time series for each group.

### Features extraction

#### Regularity dimension

The entropy measures provide a way to study the system complexity and has shown high potentiality in quantifying the extent of cortical degeneration in dementia in one of our previous studies [18]. The idea of regularity dimension is based on the concept of power laws and sample entropy (SampEn) measures [29]. SampEn quantifies the conditional probability that two sequences similar for *m *points (within a given tolerance *r*) remain similar when one consecutive point is included. For a given time series *X *= *x*_1_, *..*., *x_N_*, where *B^m^*(*r*) is the probability that two sequences will match for *m *points, whereas *A^m^*(*r*) is the probability that two sequences will match for *m *+ 1 points. However, since we normally have no a priori knowledge concerning the dimension of a system, it is imperative that we evaluate the method for different *m *and *r*. The regularity dimension has been recently introduced for modeling a mathematical relationship between the frequency with which information about signal regularity changes in various scales. It provides an approach to unify multiple solutions due to the choice among the varieties of the values of entropy parameters *m *and *r*. It can be generally expressed as where *I_r _*is SampEn denoting the information subject to *r*. It can be noted from (2) that the regularity dimension *D_r _*measures the rate of change of signal regularity/predictability with respect to log(1*/r*). It is the rate at which the entropy of a dynamical system is gained with decreasing length *r*.

(1) $$SampEn\left(m,r,N\right)=-\text{ln}\left[\frac{A^{m}\left(r\right)}{B^{m}\left(r\right)}\right]$$

(2) $$D_{r}=\;\underset{r\to 0}{\text{lim}}\frac{I_{r}}{\text{log(1}/r\text{)}}$$

**Figure 3:**
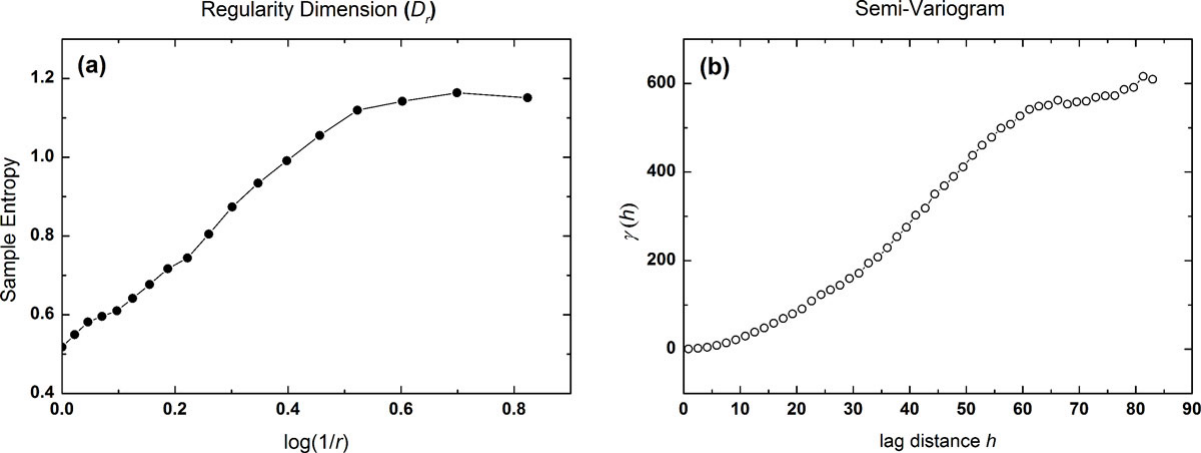
**A typical example of regularity dimension and semi-variogram**. A typical example of (a) regularity dimension and (b) semi-variogram.

In the present study, regularity dimension *D_r _*was estimated with a *r *reduced from 1 to 0.05 with an interval of 0.05. Time-series were extracted from each slice of brain MRI, thus, for each slice, we can get a feature vector consisting of *D_r _*with increasing *r *(Figure 3(a)).

#### Semi-variogram

The variogram is originally a geostatistical method that describes how the spatial data are related (correlated) with distance. It constructs a variogram that best estimates the autocorrelation structure of the underlying stochastic process. In the current study, since regularity dimension analysis only includes the information of distance values from boundary points to the gray matter center, as supplement, semi-variogram was applied to studies the spatial structure of the distance series. It includes the information of both distances and spatial locations of the boundary points. We applied the experimental semi-variogram [30], denoted as *γ*(*h*), to analyse the spatial (autocorrelation) structure of the data using a plot of the semi-variance against lag distance *h*. It is defined as the average squared difference of values separated by *h*. The semi-variogram can be calculated as where *x_i _*and *x_j _*are data values from spatial locations *i *and *j *to the center of gray matter (GM) mass, respectively. *h *represents a spatial distance that separate *x_i _*and *x_j_*. *N*(*h*) is the total number of distinct data pairs of (*x_i _− x_j_*). In the current study, *x_i _*and *x_j _*are the distance values from the cortical boundary at location *i *and *j *on to the GM center, respectively. Thus, for each time series, we can get a feature vector consisted of semi-variogram values *γ*(*h*) with increasing *h *(Figure 3(b)).

(3) $$\gamma \left(h\right)=\frac{\text{1}}{\text{2}N\left(h\right)}\underset{N\left(h\right)}{\sum }{\left(x_{i}-x_{j}\right)}^{\text{2}}$$

### Hidden Markov models (HMMs)

To study the similarities or dissimilarities between a test and reference sequences, we can apply HMMs as an efficient recognition tool. An HMM is defined as a double stochastic process, composed of an underlying stochastic process (hidden states) that can only be visualized through another set of stochastic process (observable symbols). Each HMM is characterized by *λ *= (*A*, *B*, *π*), where *A *is the transition probability matrix of the hidden states, *B *denotes the emission probability matrix of the observable symbol distributed within the hidden states, and *π *is the probability of the initial distribution of the hidden states. To be specific, the following parameters need to be defined to construct an HMM:

*λ*: HMM model, *λ *= (*A*, *B*, *π*)

*N*: the number of states

*M*: the number of different observable symbols per state

*Q*: the state sequence

*Q *= (*q*_1_, *q*_2_, *..*., *q_T_*), *T *is the number of the state sequence

*O*: the observation sequence

*O *= (*o*_1_, *o*_2_, *..*., *o_T_*), *T *is the number of observations

*A*: *A *= {*a_ij_*}, *a_ij _*is the probability of state *i *transferring to state *j*

*a_ij _*= *P*(*q_t_*_+1 _= *j|q_t _*= *i*), 1 ≤ *i*, *j *≤ *N*

*B*: *B *= {*b_j_*(*k*)}, *b_j_*(*k*) is the probability of the *k^th ^*symbol being in the state *j*

*b_j_*(*k*) = *P*(*o_t _*= *k|q_t _*= *j*), *i *≤ *j *≤ *N*, 1 ≤ *k *≤ *M*

*π*: *π *= {*π_i_*}, *π_i _*is the initial distribution of state *i*

*π_i _*= *P*(*q*_1 _= *i*), 1 ≤ *i *≤ *N*

In a hidden Markov model, the state is not directly visible, but output, dependent on the state, is visible (observable symbols). We have proved the capacity of regularity dimension as a sensitive indicator to reflect the extent of cortical degeneration in [18], so we define regularity dimension as hidden state. Semi-variogram studies the spatial structure of the time series. Since the spatial location of each point on the cortical outer boundary is observable, the semi-variogram is assumed as observable symbol. The probability of transition distribution of the states then can be estimated by states sequence, and the emission distribution probability can be estimated by observing semi-variograms where are emitted at the states of regularity dimension. The probability distribution of initial state is assumed to be as equal (0.5).

### Features coding

Since the size of feature vectors is too large for hidden Markov modelling (around 20,000 in two groups, 130-140/individual), a VQ method [31] is required to map the set of vectors into a finite, smaller, set of prototype vectors for HMM. The output VQ indexes were then applied as states or observable symbols in HMM. VQ is an efficient technique for data compression which can greatly reduce the storage and increase computational efficiency without loosing too much information. The VQ process includes two steps [32]: to design a representative codebook that minimizes the expected distortion, and assign a label (index) to each feature vector of the input data from the codebook. A most commonly used method for generating codebook is the Linde-Buzo-Gray (LBG) algorithm [33]. In general, for a given training set **T (**regularity dimension or semi-variogram in this study) and the size *J *of codebook, LBG repeatedly splits the training data into two cells until the desired size of codebook is reached. During each splitting process, search for and update the centroid of each cell until the average distortion *D *is minimized. *D *is defined by

(4) $$D=\frac{1}{TK}\overset{T}{\underset{t=\text{1}}{\sum }}{\left(||y_{t}-Q\left(x_{t}\right)|{|}_{\text{2}}\right)}^{\text{2}}.$$

**Figure 4:**
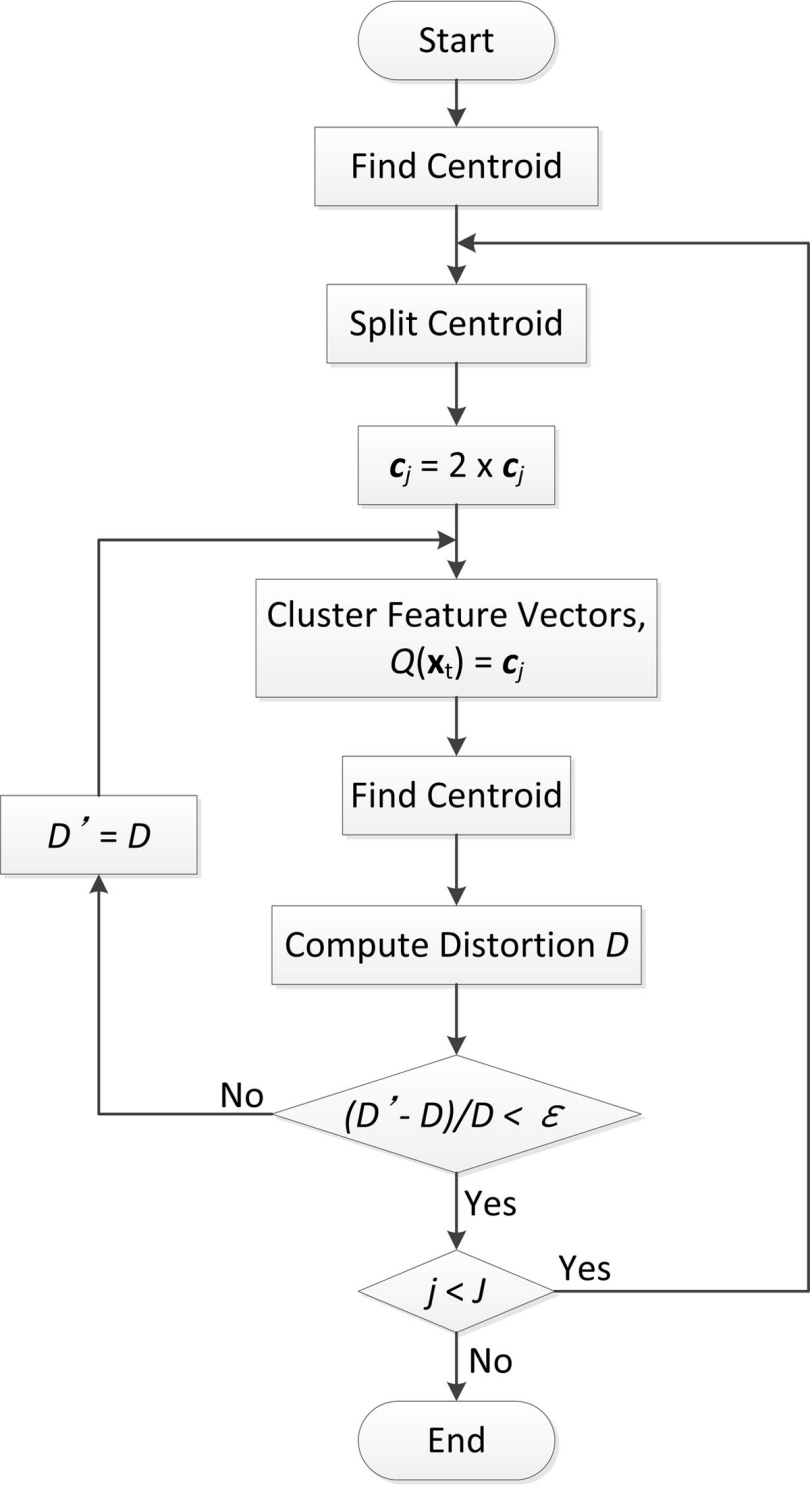
**The flowchart of LBG algorithm**. The flowchart of Linde-Buzo-Gray (LBG) algorithm.

For a training set **T **= {**y**_1_, **y**_2_,..., **y***_T _*}, where **y***_T _*= (*y_t_*_1_, *y_t_*_2_, *..*., *y_tK _*) is a *K*-dimensional feature vector, *t *= 1, 2, *..*., *T *, we aimed to find a codebook vector **C **= {**c**_1_, **c**_2_, *..*., **c***_J_*} and the partitions of space, V = {*R*_1_, *R*_2_, *..*., *R_J_*}, where *R_j _*is the encoding region associated with code vector *c_j_*, which minimize *D*. Then, each source vector **y***_t _*is assigned to a nearest neighbor encoding region *R_j _*denoted by *Q*(**x***_t_*) = **c***_j _*and labeled by index of the code vector. Only the indices are sent instead of vectors. Figure 4 shows the flowchart of LBG algorithm.

**Figure 5:**
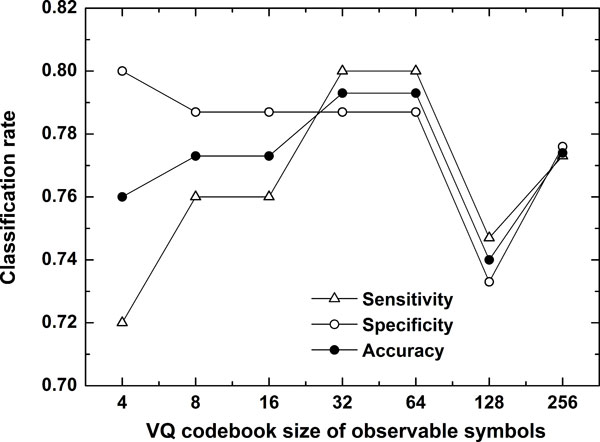
**Classification performance using fixed status size and varied sizes of observation symbols**. Classification accuracy, sensitivity and specificity of AD elders versus normal elders with a fixed VQ codebook size of states as 2 and varied VQ codebook sizes of observable symbols from 2 to 256.

In the current study, the training vectors are assembled from two groups to be compared in order to keep accordance from subject to subject. For state construction using regularity dimension, we set the size of codebook as 2. For symbol construction using semi-variogram, in order to achieve an "optimal" codebook size, we varied the size from 4 to 256. Figure 5 presents the classification rate of AD elders vs. normal elders using a fixed VQ codebook size of states as 2 and varied VQ codebook sizes for observable symbols. From the Figure 5, it can be observed that both accuracy and sensitivity improve as the size increases from 4 until 32. The accuracy as well as the sensitivity and specificity begin to decrease as the size rises to 128. The optimal size for symbols is then decided as 32 because it could provide the best trade-off between effective analysis and efficient computation.

### Similarity comparisons

For a test observation sequence *O*, representing a single individual, and a reference HMM *λ*, we can compute *P*(*O|λ*), which implies the probability of an observation sequence *O *given the model *λ*:

(5) $$\begin{array}{c} P\left(O|\lambda \right)=\underset{Q}{\sum }P\left(O|Q,\lambda \right)P\left(Q|\lambda \right)\\ \;\;\;\;\;\;\;\;\;\;\;\;\;\;\;\;\;\;\;\;\;\;\;\;\;\;\;\;\;\;\;\;\;\;\;\;\;\;\;\;\;\;\;\;\;\;\;=\underset{q_{\text{1}}...q_{T}}{\sum }\pi_{q_{1}}b_{q_{1}}\left(o_{\text{1}}\right){a_{q_{1}}}_{q_{2}}b_{q_{2}}\left(o_{\text{2}}\right)...{a_{q_{T-1}}}_{q_{T}}b_{q_{T}}\left(o_{T}\right). \end{array}$$

The time needed to evaluate *P*(*O|λ*) directly would be exponential to the observation number *T*. A forward algorithm [34] is a more efficient procedure which reduces the complexity of the calculation from 2*TN^T ^*to *N*^2^*T*.

*P*(*O|λ*) can be maximized by using Baum-Welch algorithm [23] which is also refereed to as the forward-backward algorithm. The algorithm iteratively uses $\bar{\lambda }=\left(\bar{A},\;\bar{B},\;\bar{\pi }\right)$ instead of *λ *= (*A*, *B*, *π*) to repeat the re-estimation process. The *P*(*O|λ*) improves until some limiting point is reached. The final estimate is called maximum likelihood of the HMM. The re-estimated model $\bar{\lambda }=\left(\bar{A},\;\bar{B},\;\bar{\pi }\right)$ is better than or equal to the previous model, so that $P\left(O|\bar{\lambda }\right)\geq P\left(O|\lambda \right)$, as desired.

Given a test HMM *λ*_1 _= (*A*_1_, *B*_1_, *π*_1_) and a reference HMM *λ*_2 _= (*A*_2_, *B*_2_, *π*_2_), we can compare the similarity/dissimilarity between the two models by using a well-known proximity measure, Kullback-Leibler divergence (KLD) [35]. The KLD estimate two probability distributions between two HMMs *λ*1 and *λ*2 which can be defined by

(6) $$D_{KL}\left(\lambda_{\text{1}},\lambda_{\text{2}}\right)=\text{1}-\text{exp}\left[D_{s}\left(\lambda_{\text{1}},\lambda_{\text{2}}\right)\right],$$

where *D_s _*is the symmetrized version of the approximate KLD of *λ*_1 _and *λ*_2_, namely

(7) $$D_{s}\left(\lambda_{\text{1}},\lambda_{\text{2}}\right)=\frac{D\left(\lambda_{\text{1}},\lambda_{\text{2}}\right)+D\left(\lambda_{\text{2}},\lambda_{\text{1}}\right)}{2},$$

in which *D*(*λ*_1_, *λ*_2_) is the empirical KLD between *λ*_1 _and *λ*_2 _which was originally introduced by Juang and Rabiner [32] using the Monte Carlo simulations. The models are assumed to be ergodic, having arbitrary observation probability distributions and the dissimilarity is defined as the mean divergence of the observation sample. This approximate KLD is given by where $O_{\lambda_{2}}=\left(o_{1},o_{2},\ldots ,o_{T_{2}}\right)$ is a sequence of observations generated by model *λ*_2_, and *T*_2 _is the length of the sequence $O_{\lambda_{2}}$. Eq. (8) can interpret how well model *λ*_1 _matches observations generated by model *λ*_2_, relative to how well model *λ*_2 _matches observations generated by itself.

(8) $$D\left(\lambda_{\text{1}},\lambda_{\text{2}}\right)\text{ = }\frac{1}{T_{2}}\text{log }\frac{P\left(O_{\lambda \text{2}}|\lambda_{\text{1}}\right)}{P\left(O_{\lambda \text{2}}|\lambda_{2}\right)},$$

To be symmetric, we define *D *(*λ*_2_, *λ*_1_) as where $O_{\lambda_{1}}=\left(o_{1},o_{2},\ldots ,o_{T_{1}}\right)$ is a sequence of observations generated by model *λ*_1_, and *T*_1 _is the length of the sequence $O_{\lambda_{2}}$.

(9) $$D\left(\lambda_{\text{2}},\lambda_{\text{1}}\right)\text{ = }\frac{1}{T_{1}}\text{log }\frac{P\left(O_{\lambda 1}|\lambda_{2}\right)}{P\left(O_{\lambda 1}|\lambda_{1}\right)},$$

### HMM implementation

The implementation of the brain HMM is outlined as follows:

1) Obtain MRI scans of a participant;

2) Extract grey matter from pre-segmented image using SPM software package (http://www.fil.ion.ucl.ac.uk/spm);

3) Extract time series by the distances measured from subsequent outer boundary points to the GM center of mass;

4) Extract state and observable symbol sequences based on regularity dimension and semi-variogram using the VQ codebook;

5) Initial estimate *λ *= (*A*, *B*, *π*);

6) Re-estimate $\bar{\lambda }=\left(\bar{A},\;\bar{B},\;\bar{\pi }\right)$ using Baum-Welch algorithm.

### HMM testing

The classifier was tested between every two groups (elder AD vs. elder non-demented, middle-aged and young, respectively) using leave-one-out (LOO) and n-fold cross-validation. In LOO method, one subject is taken at a time for evaluation, and the remaining subjects are taken for training. This process is repeated until each subject in both groups has been taken for evaluation, yielding an unbiased estimate of the classification error rate. In n-fold cross-validation method, all the datasets are divided into training and testing dataset. Firstly, 50% of the dataset are randomly selected as the training set, while the rest 50% are taken for testing. The mean classification rates were obtained by repeating the testing process over 100 times. Next, we increase the traning data to 70% and 90% and test the models on the rest 30% and 10% data, respectively. For each combinations, the testing process was repeated over 100 times to get mean classification rates.

To quantify the classification results, we calculated the accuracy defined as the ratio of the number of test subjects correctly classified to the group. Meanwhile, sensitivity and specificity of each tested pair of groups were computed as: *Sensitivity *= *TP/*(*TP *+ *FN*) and *Specificity *= *TN/*(*TN *+ *FP*), where true positives (TP) are number of demented patients correctly classified; true negatives (TN) are the number of control subjects correctly classified; false positives (FP) are the number of controls classified as demented patients and false negatives (FN) are the number of demented patients classified as normal controls.

## Results and Discussion

The testing results of HMM classifier are shown in Table 2. From Table 2, it is noted that the classification rate increased as the age difference becomes larger. This is accordance with our previous finding of an age-related progressing in cortical structural irregularity [18]. Normal aging can also result in a reduction in brain size. Although AD may exhibit a characteristic pattern of atrophy different from that due to aging, even accelerated aging, when AD works as an added effect over aging, it is difficult to ascertain whether the cognitive decline is simply resulted from normal aging or AD, especially at early stage of the disease. This is why the classification accuracy is relatively lower in AD elders versus normal elders. When without this added effect, the accuracy rises (in LOO, from 80.7% to 98.7%) as well as the sensitivity (in LOO, from 81.3% to 98.7%) and specificity (in LOO, from 80.0% to 98.7%). Results from the 50%, 70% and 90% training sets showed that the larger the training set is, the higher the mean classification rates. However, two testing approaches using *P*(*O|λ*) and KLD don't differ in classification results (Table 3). Since KLD estimation is based on *P*(*O|λ*), a single use of *P*(*O|λ*) is superior to KLD which provides with more efficient computing. Meanwhile, re-estimation of HMM parameters did not improve the classification results as shown in Table 4. One of the possible reasons could be a small size of training data that allows in parameter re-estimation. Another reason may be while parameters of one HMM are updated in such a way to maximize the quantity *P*(*O|λ*), the probability of *O *being observed from the other model is also improved. For those *P*(*O|λ*) of one group HMM only a tiniest bit over that relative to the other group HMM, there is a risk that the re-estimated parameters may lead to a completely converse classification result.

**Table 2:** Testing Results Using N-fold and Leave-one-out Cross-validation

Training Sets	Parameters	AD vs O (mean *± *sd)	AD vs M (mean *± *sd)	AD vs Y (mean *± *sd)
50%	Sensitivity	0.792 *± *0.087	0.933 *± *0.033	0.982 *± *0.020
	Specificity	0.773 *± *0.070	0.900 *± *0.047	0.982 *± *0.019
	Accuracy	0.782 *± *0.047	0.916 *± *0.026	0.982 *± *0.013
70%	Sensitivity	0.790 *± *0.087	0.934 *± *0.045	0.986 *± *0.025
	Specificity	0.076 *± *0.084	0.891 *± *0.055	0.982 *± *0.023
	Accuracy	0.783 *± *0.053	0.912 *± *0.035	0.984 *± *0.015
90%	Sensitivity	0.780 *± *0.169	0.934 *± *0.090	0.986 *± *0.048
	Specificity	0.800 *± *0.163	0.891 *± *0.117	0.980 *± *0.050
	Accuracy	0.790 *± *0.117	0.913 *± *0.065	0.983 *± *0.035
Leave-one-out	Sensitivity	0.813	0.933	0.987
	Specificity	0.800	0.920	0.987
	Accuracy	0.807	0.927	0.987

AD, O, M, Y refer to elder AD, non-demented elder, middle-aged, and young, respectively

**Table 3:** Classification Rates Using *P*(*O|λ*) and KLD

	Sensitivity		Specificity		Accuracy	
	
	*P*(*O|λ*)	KLD	*P*(*O|λ*)	KLD	*P*(*O|λ*)	KLD
AD vs O	0.813	0.813	0.800	0.800	0.807	0.807
AD vs M	0.933	0.933	0.920	0.920	0.927	0.927
AD vs Y	0.987	0.987	0.987	0.987	0.987	0.987

**Table 4:** Classification Rates Using Initial and Re-estimated HMMs

		Sensitivity		Specificity		Accuracy	
	
		*λ*	$\lambda^{\prime }$	*Λ*	$\lambda^{\prime }$	*λ*	$\lambda^{\prime }$
AD vs O	*P*(*O|λ*)	0.813	0.667	0.800	0.640	0.807	0.653
	KLD	0.813	0.667	0.800	0.640	0.807	0.653

*λ *and $\lambda^{\prime }$ refer to initial and re-estimated HMMs, respectively

Using the aforementioned experimental methods, demented images can be classified into three categories: very mild, mild and moderate as shown in Table 5. The resulting detection rate of dementia increased as the cognitive impairment exacerbated as expected (Table 6). The declination of global GM volume accelerates as disease developed [6] in the sense the morphological abnormalities can be even more remarkable which make them more likely to be predicted and differentiated from normal cognitive decline. However, due to the limitation of the data provided by OASIS database which include very few moderate AD samples, even the current results show a very encouraging detection rate in latter two groups, we are not able to verify the performance of the classifier in late AD recognition. This issue is certainly worth investigating in our future research when more data become available.

**Table 5:** Subjects Dementia Status

	Very Mild	Mild	Moderate
Number	52	21	2
Age (years)	76.8 *± *7.8	78.4 *± *7.3	82.0 *± *5.6
CDR	0.5	1	2
Mini Mental State Examination (MMSE)	25.1 (14*∼*30)	22.2 (15*∼*29)	15

**Table 6:** Changes of Detection Rate as Disease Developed

	CDR	Detection Rate
Very mild	0.5	0.769
Mild	1	0.905
Moderate	2	1.000

We have demonstrated that our proposed HMMs for MRI data analysis has strong potential in discriminating among early AD, and healthy controls. We also compared our classification results with recent related works using the same OASIS database. Garcia-Sebastian et al. [36] applied voxel-based morphometry (VBM) to extract classification features and SVM algorithm to perform classification of patients with mild AD (49) vs. controls (49). They obtained a better results with 87.5%. Savio et al. [37] applied four different models of ANN to the same dataset using SVM, and reported a best classification accuracy of 83%. Comparing to these studies that achieved higher accuracies but using small sampler sizes, our classification result of 80.7% accuracy is still encouraging considering the number of subjects in the database and presented more statistically reliable results. Daliri [38] proposed an automated method using scale-invariant feature transform (SIFT) and SVM for diagnosing AD. Study achieved a classification accuracy of 86% for mild AD (20) vs. normal (66) and 75% for (very mild + mild AD) (69) vs. normal (66). The author also noted that it is more difficult to classify the data from the subjects with very mild AD or the data from the subjects that are elderly. Another study reported by Zhou et al. [39] using a large sample size proposed a framework to analyze the hippocampal shape difference between AD (85) and HC subjects (79) and achieved a classification accuracy of 52.6 *∼ *61.5%. Yang et al. [40] proposed a method based on independent component analysis (ICA) coupled with the use of SVM for classifying MRI scans into categories of AD (100), young healthy controls (yHC) (116), middle age healthy controls (mHC) (100) and old healthy controls (oHC) (100). The best classification accuracy between AD and oHC, AD and mHC, AD and yHC appeared to be 73.7%, 91.6% and 97.8%, respectively. Comparing to above studies using large samples, our results show better accuracy as well as sensitivity and specificity.

HMMs have been applied to audio recognition and gesture/motion detection for monitoring daily activities of patients with dementia [41-43]. It has been also utilized to brain MRI for age prediction [25,26]. To our knowledge, there has been no report on the application of HMMs in dementia classification. We explored the usage of HMM and proved its potentiality in early AD diagnosis. The results from the 50%, 70% and 90% training sets show that larger training set did achieve better accuracy but did not significantly improve the classification performance. It suggests that only a small training set (50%) is large enough for HMMs. Comparing to conventional modeling method using SVM or relevance vector regression (RVR) that usually needs for large training data and effective feature selection which is sometimes difficult to give clinical interpretations, training and validation on the extracted features using HMM can be more time efficient. Meanwhile, the superiority of HMMs in sequence statistics enables the sequence information of brain MRI slices in representing the global cortical structure which makes the results favorable to clinical analysis.

Our main goal in the current study is to propose a method based on HMM modeling for dementia recognition. The current results have shown the capability of our proposed method for early disease diagnosis. Since it is a pilot study, the current classification results are encouraging and reasonable but still can be improved. We have noticed that some white matter (WM) hyper-intensities were classified as GM, especially the boarder voxels between GM and WM, and possibly the GM center of mass may be influenced by the presence of the WM hyper-intensities. Due to the limitation of the segmentation and boundary tracing method, some potential sensitivity of the approach is probably lost. Future study will focus on methodology refinements to achieve a higher classification accuracy especially between AD elders and normal elders. Moreover, we aim to extent the use of this modeling to longitudinal dataset provided by OASIS database, and also to larger datasets such as that provided by Alzheimer's Disease Neuroimaging Initiative (ADNI). We are also interested to know if our proposed model can classify between AD and other neurological diseases.

## Conclusion

We have presented an approach for modeling MRI based whole brain structural complexity and applied it for diagnosing Alzheimer disease. Comparison with other related work using the same OASIS database, we found the classification performance of our proposed model presents promising results in classification. These results demonstrate that our proposed approach is potential to serve as an in vivo surrogate tool for disease severity prediction, and as a diagnostic method for mild cognitive impairment and AD.

## Competing interests

The authors declare that they have no competing interests.

## Authors' contributions

YC conducted the study, evaluated the data, performed data analyses, and drafted the manuscript. TP conceived and supervised the study, revised, and approved the final manuscript.
